# Changes in enteric fever trends during the COVID-19 pandemic from the Surveillance for Enteric Fever in Asia Project: a cross-sectional study

**DOI:** 10.1016/j.lansea.2025.100562

**Published:** 2025-03-29

**Authors:** Sira Jam Munira, Shiva R. Naga, Irum Fatima Dehraj, Kate Doyle, Naito Kanon, Mohammad Tahir Yousafzai, Dipesh Tamrakar, Afshan Piyar Ali, Annaya Barman Jui, Alice S. Carter, Dipu Chandra Das, Surrendar Dawani, Khalid Iqbal, Seema Irfan, Mohammad Shahidul Islam, Muhammad Ashraf Memon, Tuba Khan, Shamsun Nahar, Md. Hafizur Rahman, Nasir Saleem Saddal, Jessica C. Seidman, Rajeev Shrestha, Humaira Susmita, Jason R. Andrews, Stephen P. Luby, Denise O. Garrett, Farah Naz Qamar, Samir K. Saha, Senjuti Saha

**Affiliations:** a Child Health Research Foundation, Dhaka, Bangladesh; b Dhulikhel Hospital, Kavrepalanchok, Nepal; c Kathmandu University Hospital, Kathmandu, Nepal; d Department of Pediatrics and Child Health, Aga Khan University, Karachi, Pakistan; e Sabin Vaccine Institute, Washington, DC, USA; f Aga Khan University, Karachi, Pakistan; g Jinnah Postgraduate Medical Center, Karachi, Pakistan; h Clinical Laboratory, Kharadar General Hospital, Karachi, Pakistan; i Department of Pathology and Laboratory Medicine, Aga Khan University, Karachi, Pakistan; j Kharadar General Hospital, Karachi, Pakistan; k National Institute of Child Health, Karachi, Pakistan; l Center for Infectious Disease Research & Surveillance, Dhulikhel Hospital, Kavrepalanchok, Nepal; m Research & Development Division, Dhulikhel Hospital, Kavrepalanchok, Nepal; n Division of Infectious Diseases and Geographic Medicine, Stanford University, Stanford, CA, USA

**Keywords:** Typhoid, Paratyphoid, Enteric fever, Southeast Asia, Typhi, Paratyphi, Surveillance, Antimicrobial resistance, Bangladesh, Nepal, Pakistan, Covid19 pandemic

## Abstract

**Background:**

The Surveillance for Enteric Fever in Asia Project (SEAP) conducted blood culture surveillance for *Salmonella enterica* serotype Typhi (*S*. Typhi) and Paratyphi (*S*. Paratyphi) to provide an evidence base for prevention and control measures in Bangladesh, Nepal, and Pakistan.

**Methods:**

From October 2020 to September 2022, we conducted prospective clinical surveillance and retrospective laboratory surveillance at health facilities in Dhaka, Bangladesh; Kathmandu and Kavrepalanchok, Nepal; and Karachi, Pakistan. Patients were eligible if they were outpatients with three or more days of fever in the last week. In Nepal and Pakistan, inpatients were eligible if they had suspected or confirmed enteric fever; in Bangladesh, only inpatients with confirmed enteric fever were enrolled. Patients with blood culture–confirmed enteric fever identified by hospital laboratories and laboratory network sites were also enrolled. Patients completed interviews and medical records were reviewed and abstracted. All enrolled patients had blood cultures performed. Antibiograms were performed to characterize drug sensitivity. We summarized the data descriptively.

**Findings:**

A total of 17,593 patients were enrolled from 19 facilities. Of these, 8410 patients had culture-confirmed enteric fever. Case counts in all countries decreased in the early stages of the COVID-19 pandemic, but increased over time in Bangladesh and Pakistan. Case counts remained low throughout the study period in Nepal. In all countries, typhoid was more common than paratyphoid; the proportion of paratyphoid cases ranged from 8.4% in Pakistan to 16% in Nepal. Extensively drug-resistant typhoid was common in Pakistan (69%), but was not detected in Bangladesh or Nepal.

**Interpretation:**

Cases of enteric fever decreased during the COVID-19 pandemic, though it is not clear how much of this decrease relates to true changes in transmission versus health-seeking behavior.

**Funding:**

This project was funded by the 10.13039/100000865Gates Foundation through INV-008335.

**Research in context**

**Evidence before this study**

We did an extensive literature search on PubMed, Google Scholars, bioRxiv, and medRxiv using the key terms “Enteric fever”, “Salmonella Typhi”, “Salmonella Paratyphi”, “Antimicrobial resistance”, “COVID-19 AND Enteric fever”, “COVID-19 AND Disease surveillance” available as of August 14, 2023, published in English. We also examined evidence pertaining to the disease burden and patterns of antimicrobial resistance (AMR) of enteric fever within the context of ongoing pandemic. The Phase II of Surveillance of Enteric Fever in Asia Project (SEAP) has already reported the incidence data on typhoid and paratyphoid in Bangladesh, Nepal, and Pakistan. Several other studies have explored the disease burden, risk factors, case fatality, and AMR pattern of typhoid and paratyphoid fever before the COVID-19 pandemic period. However, no prior studies have documented the effects of COVID-19 pandemic on the observed burden of enteric fever and AMR pattern within the ongoing febrile surveillance.

**Added value of this study**

This study yielded significant insights into the impact of COVID-19 pandemic on an enteric fever surveillance initiative. It also investigated any altered dynamics of enteric fever disease trend following the onsets of the pandemic. Despite the challenges posed by pandemic, our study revealed a persistent high burden of enteric fever in Bangladesh, Pakistan, and Nepal. Furthermore, we scrutinized any shifts in AMR pattern for both typhoid and paratyphoid fever during the pandemic period.

**Implications of all the available evidence**

The ongoing challenges posed by enteric fever coupled with the evolving antimicrobial resistance pattern serve as powerful evidence, supporting the urgent need for improved access to vaccination in Nepal and Pakistan, as well as timely introduction of TCV in Bangladesh. Continued data collection is essential for assessing the long-term effects of TCV on disease occurrence and prevalence of antimicrobial resistance. Such data is crucial not only for enhancing vaccination accessibility but also for prompt identification of any emerging resistance trends, and effectively mitigating the impact of antimicrobial resistance.

## Introduction

Enteric fever, caused by *Salmonella* Typhi or Paratyphi, is primarily transmitted through fecal-oral routes and mainly occurs in low and middle-income countries.^1^ In 2019, there were an estimated 91.2 million cases with 110,000 associated deaths of enteric fever worldwide.^2^ The highest incidence is in South Asia and among children aged 5–9 years and adolescents of 10–14 years.^3^ Antimicrobial-resistant (AMR) strains reduce treatment efficacy, increase the cost of clinical management, and ultimately exacerbate the mortality and morbidity of enteric fever.^4^

In 2018, the World Health Organization (WHO) recommended introducing typhoid conjugate vaccine (TCV) in all high typhoid burden areas.^5^ A single dose of TCV has demonstrated high safety and efficacy (78·3%) among children aged 9 months–12 years; 163 children needed to be vaccinated to prevent one case.^6^ Recently published data shows vaccine protection for four years for Malawian children and two years for Bangladeshi children.^6^^,^^7^ Introducing TCV nationwide, coupled with an additional mop-up campaign, can serve as a cost-effective strategy for alleviating the burden in typhoid-endemic regions.^8^^,^^9^

The Surveillance for Enteric Fever in Asia Project (SEAP) was a multiphase blood culture-based active surveillance program. In phase I (2014–2016), SEAP conducted a retrospective record review to generate data on the enteric fever burden at sites in Bangladesh, India, Nepal, and Pakistan.^10^ In phase II (2016–2019), SEAP established prospective blood-culture surveillance at partner hospitals and a network of diagnostic centers in Bangladesh, Nepal and Pakistan.^11^ Detailed findings regarding incidence estimates, illness severity, antimicrobial resistance, healthcare utilization patterns, and the economic burden of illness from prior years were published previously.^12^ These data supported government decisions to introduce TCV into national immunization schedules in Pakistan (2019), Nepal (2022), and Bangladesh (planning introduction). Updated and comprehensive surveillance data on high burden areas is helpful to inform selection of target populations, optimal age groups, and effective delivery strategies during introduction of vaccines into routine immunization programs.

Nepal introduced the typhoid conjugate vaccine into routine immunization in April 2022. The introduction began with a catch-up campaign for all children aged 15 months through 15 years; campaign coverage was reported as over 99%.^13^ In Pakistan, TCV is also in routine use.^14^ The vaccine was introduced in phases: first, a catchup campaign for children 9 months to 15 years in urban Sindh (including our study catchment area) in November 2019, followed by routine introduction for children 9 months old throughout Sindh during January 2020. A recent study from Pakistan reported a steep decline in overall immunization coverage during the pandemic compared to the pre-pandemic period.^15^

Phase III of SEAP surveillance, which started in October 2019, continued health facility-based enrollment of febrile patients to track enteric fever disease burden, illness severity, and AMR patterns. Shortly after the start of SEAP III, the COVID-19 pandemic started. The WHO officially declared a global public health emergency on 30 January 2020.^16^ In the SEAP countries, the first COVID-19 case was detected on 23 January 2020 in Nepal, 26 February 2020 in Pakistan, and 8 March 2020 in Bangladesh.^17^ Large-scale public health measures were implemented to combat transmission, including stay-at-home orders, lockdowns, and healthcare changes, such as establishing fever clinics or changing patient pathways in clinical centers. Routine healthcare appointments were postponed or canceled, and people were hesitant to seek medical attention for non-serious health issues due to fear of exposure to the virus, societal anxiety, or concerns about overwhelmed healthcare systems.^18^ Limited access to proper care, changes in healthcare-seeking behaviours, and improper antibiotics usage had a substantial impact on overall observed disease trends, healthcare systems, and ongoing disease surveillance.^19^

This paper describes the patterns of enteric fever and antimicrobial resistance in three countries during an acute period of the pandemic. Our surveillance provides early context for the impacts of the COVID-19 pandemic on active febrile surveillance studies and can inform local implementation of enteric fever prevention measures, including vaccines.

## Methods

### Study design

In this cross-sectional study, we conducted enteric fever surveillance in 19 health facilities from October 2019 to September 2022. Prospective surveillance of febrile patients was conducted at six tertiary hospitals (two in each country). Both hospitals in Bangladesh serve pediatric patients; all other hospitals see patients of all ages. We also retrospectively enrolled culture-confirmed enteric fever patients identified by the laboratories at these hospitals and at 13 additional diagnostic laboratory sites (see Appendix for full list of facilities.) The criteria for selecting the active clinical surveillance sites were described previously.^12^

Individual eligibility and recruitment criteria varied by site, and there were some changes from previous reports. Outpatients in Nepal and Pakistan were offered enrollment if they reported three or more consecutive days of fever in the last seven days and lived in the pre-defined catchment area. In Bangladesh, outpatients who met these eligibility criteria were screened, and offered enrollment if they were culture-positive. Inpatients in Nepal and Pakistan were enrolled if they had suspected or confirmed enteric fever; in Bangladesh, inpatients were only enrolled if they had culture-confirmed enteric fever. Additionally, at all study sites, culture-confirmed enteric fever cases from the hospital laboratories and laboratory network facilities were offered enrollment. At enrollment, research staff reviewed patient medical records and completed questionnaires on signs and symptoms. In Nepal and Pakistan, all cases received follow-up calls six weeks after positive culture to collect information on relapse, morbidity, and mortality. In Bangladesh, all paratyphoid cases and a randomly selected 25% of positive typhoid cases received follow-up calls.

### Ethical procedures

All participants provided written informed consent prior to study enrollment. For participants under 18 years, parents or guardians provided informed consent. In Bangladesh, children aged 15–17 years provided an additional written assent. This study received ethical approval from institutional review boards in the USA (Stanford University Institutional Review Board #39557), Bangladesh (Ethical Review Committee of Bangladesh Shishu Hospital and Institute, BCH-ERC-24-04-21 (01)), Nepal (Nepal Health Research Council and Institutional Review Committee 391/2018, Kathmandu University School of Medical Sciences 56/19), and Pakistan (AKUH Ethics Review Committee ref 2019-0410-2861, Pakistan National Bioethics Committee no. 4-87/NBC-341-Amend/19/81).

### Laboratory and statistical methods

Laboratory methods were described previously.^12^ Briefly, in all laboratories, whole blood was incubated in BACTEC™ automated culture systems (Becton Dickinson, Franklin Lakes, NJ, USA) and positive samples were subcultured on agar plates to see the morphology of the bacteria. Species were confirmed using biochemical testing. Specimens from the laboratory network sites were confirmed by study hospital laboratories. Antibiotic susceptibility was assessed using disc diffusion following Clinical and Laboratory Standard Institute Guidelines-M100.^20^ Isolates were classified as multidrug-resistant (MDR) if they were resistant to ampicillin, chloramphenicol, and cotrimoxazole. Isolates were considered extensively drug-resistant (XDR) if they were multidrug-resistant as described, as well as non-susceptible to any fluoroquinolone and resistant to any third-generation cephalosporin. To maintain uniformity across study sites, all laboratories used the same culture system and conducted routine monitoring checks.

Participant data were collected through medical record review and patient interviews using a custom Android application by trained study staff. Vaccination status and symptoms were self-reported. We performed data cleaning and descriptive analyses using SAS (SAS Institute, Cary, NC, USA) and R (version 4.3.1, R Core Team, Vienna, Austria).

### Role of the funding source

The funder of the study approved the study design, but was not involved in data collection, data analysis, data interpretation, or writing of the report.

## Results

**Fig. 1:**
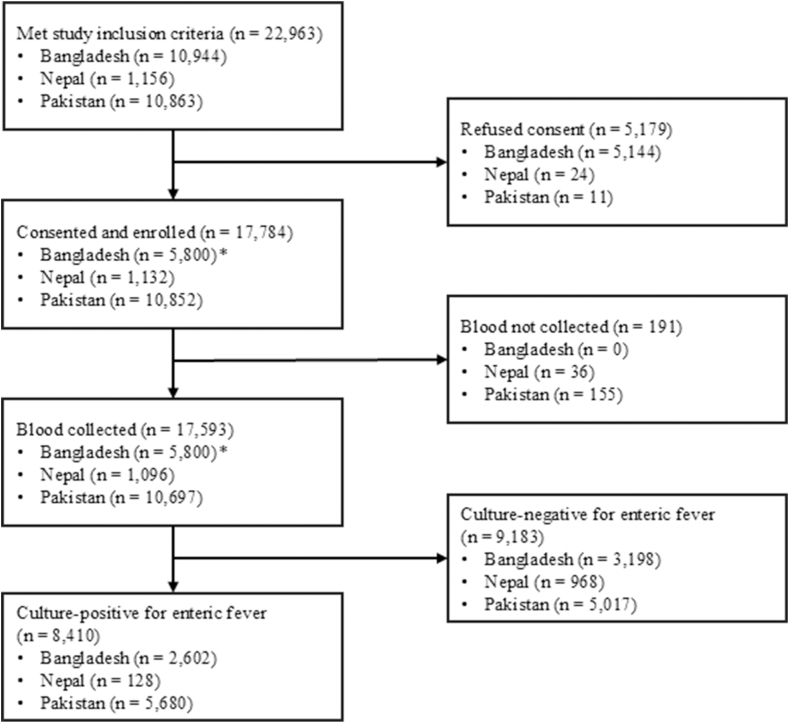
**Recruitment, eligibility, study consent, and laboratory culture positivity**. ∗Participants in Bangladesh were pre-enrolled prior to blood collection and enrolled following a positive culture.

Across all sites, between October 2019 and September 2022, 17,593 participants had blood culture performed, and 8410 were culture-confirmed enteric fever cases. There were 128 enteric fever cases enrolled in Nepal, and 5680 in Pakistan; 2602 cases were enrolled in Bangladesh, where only culture-confirmed cases were offered enrollment (Fig. 1). The age distribution of screened participants varied by country; the median age was youngest in Bangladesh (4 years [interquartile range 2–8 years]) (where recruitment is primarily at pediatric facilities), oldest in Nepal (23 years [IQR 9–39]), and quite young in Pakistan (5 years [IQR 3–14]) (Table 1). These trends were repeated among confirmed enteric fever cases, where the median age was 6 years in both Bangladesh and Pakistan, but 21 years in Nepal.

**Table 1:** Participant demographics by country.

	Bangladesh		Nepal		Pakistan	
	n	%	n	%	n	%
**All participants with culture performed**						
**Total**	5800		1096		10,697	
Sex						
Female	2517	43%	481	44%	4466	42%
Male	3283	57%	615	56%	6231	58%
Age, years (median (IQR))	4 (2, 8)		23 (9, 39)		5 (3, 14)	
Age group						
<5 years	2984	51%	147	13%	4329	40%
5–14 years	2342	40%	197	18%	3708	35%
15–44 years	455	7.8%	524	48%	2070	19%
45–64 years	17	0.3%	168	15%	422	3.9%
65+ years	2	<0.1%	60	5.5%	168	1.6%
Study recruitment location						
Outpatient department	3720	64%	873	80%	2611	27%
Inpatient department	500	8.6%	112	7%	3432	31%
Hospital laboratory	897	15%	33	3.0%	99	0.7%
Laboratory network site	683	12%	78	10%	4555	42.0%
Typhoid vaccination status						
Vaccinated	8	0.1%	77	7.0%	1547	14%
Not vaccinated	2571	44%	1009	92%	7805	73%
Unknown	3221	56%	10	0.9%	1345	13%
**Laboratory-confirmed enteric fever cases**						
**Total**	2602		128		5680	
Sex						
Female	1094	42%	60	47%	2314	41%
Male	1508	58%	68	53%	3366	59%
Age, years (median (IQR))	6 (3, 11)		21 (16, 26)		6 (3, 10)	
Age group						
<5 years	977	38%	2	1.6%	2368	42%
5–14 years	1172	45%	25	20%	2352	41%
15–44 years	434	17%	93	73%	889	16%
45–64 years	17	0.7%	7	5.5%	58	1.0%
65+ years	2	<0.1%	1	0.8%	13	0.2%
Study recruitment location						
Outpatient department	526	20%	13	10%	327	5.8%
Inpatient department	496	19%	4	3.1%	699	12%
Hospital laboratory	897	34%	33	26%	99	1.7%
Laboratory network site	683	26%	78	61%	4555	80%
Serovar						
Typhi	2220	85%	108	84%	5205	92%
Paratyphi A	382	15%	20	16%	475	8.4%
Typhoid vaccination status and serovar						
Vaccinated	8	0.3%	3	2.3%	628	11%
Vaccinated and *S.* Paratyphi A	2/8	25.0%	3/3	100.0%	72/628	11%
Vaccinated and *S.* Typhi	6/8	75.0%	0/3	0.0%	556/628	89%
Not vaccinated	2563	99%	122	95%	4130	73%
Unknown	31	1.2%	3	2.3%	922	16%

Among culture-confirmed enteric fever cases, the majority were *S.* Typhi (Bangladesh, 85% [2220/2602]; Nepal, 84% [108/128]; Pakistan, 92% [5205/5680]) (Table 1). Among cases, typhoid vaccination (self-reported) was rare in Bangladesh (0·8% [8/2602]) and only occurred among participants in a typhoid vaccine clinical trial. In Nepal, 2·3% (3/128) participants with enteric fever reported vaccination, though vaccination was only available during the last six months of surveillance. In Pakistan, 11% (628/5680) reported being vaccinated.

**Fig. 2:**
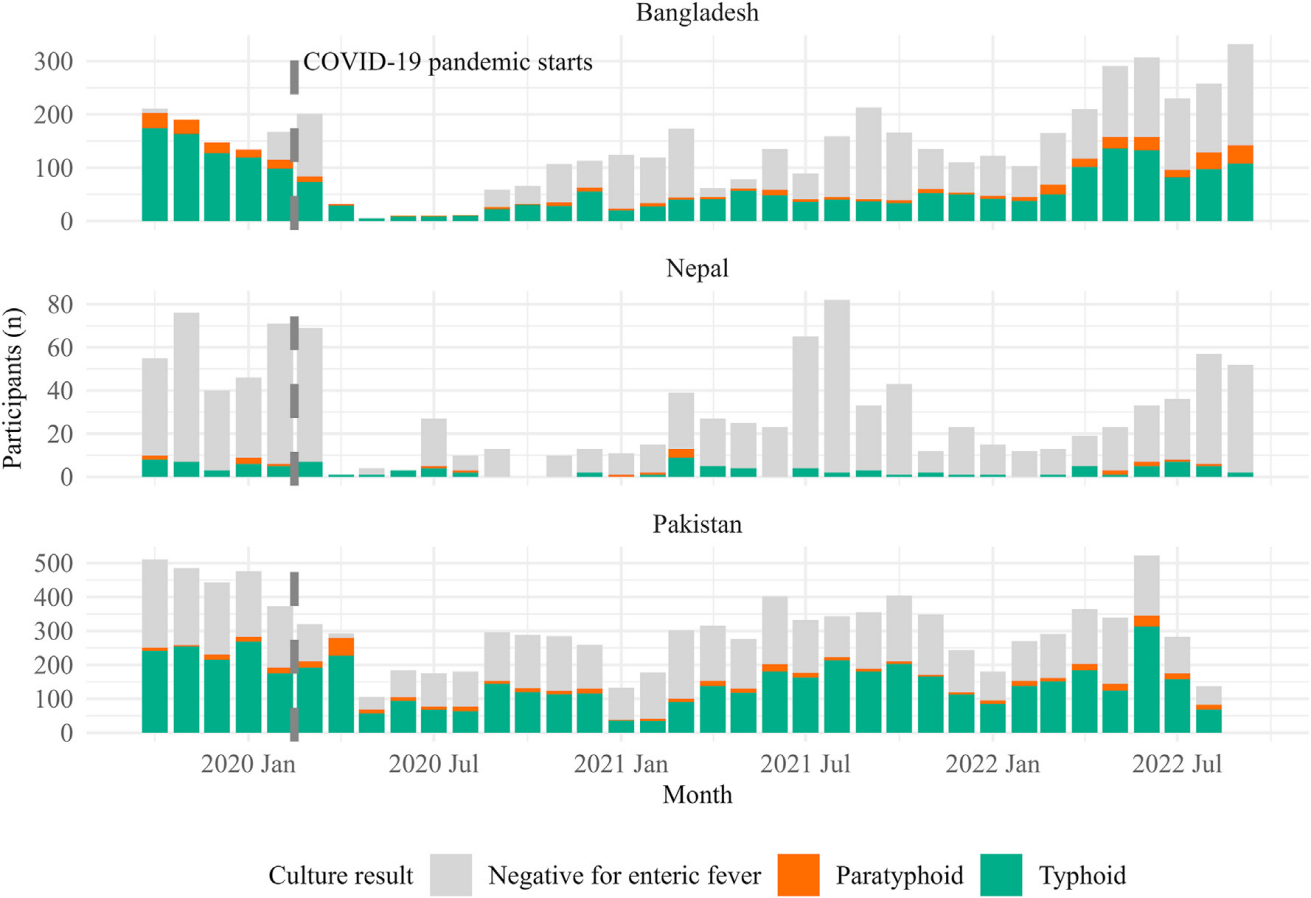
**Study participation by culture result and country**.

In Bangladesh, enrollment and screening decreased at the beginning of the project, likely due to changes in recruitment procedures, and dropped precipitously in the early stages of the pandemic (Fig. 2). In April 2020, recruitment in the study dropped 91% compared to February 2020. However, both enrollment and screening increased through 2021, and returned to pre-pandemic levels by the summer of 2022. In Nepal, enrollment similarly dropped drastically after the pandemic start; recruitment in April 2020 was 98% lower than February 2020. Study enrollment and case counts increased in 2021 and 2022. In Pakistan, enrollment dropped in the early study period, though much less than in the other countries; recruitment in April 2020 was only 21% lower compared to February 2020. Case counts and enrollment returned to previous high levels after the initial onset of the pandemic.

We described illness and impact among participants with culture-confirmed typhoid and paratyphoid fever (Table 2). Common clinical symptoms included fever (which is also part of the recruitment criteria for prospective participants), vomiting (Bangladesh, 21% [539/2602]; Nepal, 34% [44/128]; Pakistan, 38% [2174/5680]) and diarrhea (Bangladesh, 16% [427/2602]; Nepal, 26% [33/128]; Pakistan, 29% [1634/5680]). Despite similar days of fever at presentation, the number of days patients were unable to conduct normal activities differed by location: 3 days (IQR 0–7) in Bangladesh, 7 days (IQR 5–10) in Nepal, and the longest, 10 days (IQR 5–14), in Pakistan. The percent of patients who reported prior antibiotic use before coming to the health facilities was highest in Bangladesh (39% [1005/2602]), lower in Nepal (26% [33/128]), and lowest in Pakistan (20% [1113/5680]).

**Table 2:** Description of illness episode by country among participants with culture-confirmed typhoid and paratyphoid.

	Bangladesh (n = 2602)		Nepal (n = 128)		Pakistan (n = 5680)	
	n	%	n	%	n	%
**Clinical signs and symptoms**						
Fever	2599	100%	127	99%	5676	100%
Median duration of fever (IQR) in days at enrollment	5	(4, 7)	6	(4, 8)	7	(4, 10)
Vomiting	539	21%	44	34%	2174	38%
Diarrhea	427	16%	33	26%	1634	29%
Abdominal pain	598	23%	35	27%	1775	31%
Headache	469	18%	76	59%	989	18%
**Patient behavior**						
Median days not able to conduct normal activities (IQR)	3	(0, 7)	7	(5, 10)	10	(5, 14)
Received typhoid vaccine prior to illness	8	0.3%	3	2.3%	628	11%
Antibiotic use prior to enrollment visit	1005	39%	33	26%	1113	20%
**Outcomes**						
Hospitalized	712	27%	35	28%	1838	33%
Death	1	<0.1%	0	0%	5	<0.1%

The highest proportion of hospitalized laboratory-confirmed enteric fever cases was in Pakistan (33% [1838/5680]); the rate was similar in Bangladesh (27% [712/2602]) and Nepal (28% [35/128]). Six total deaths among enteric fever patients were reported in the study; five deceased patients were in Pakistan and one in Bangladesh. All deceased participants had typhoid; the median age was 1.2 years (IQR 0·9–1·9 years). For the five participants with a cause of death listed (one cause of death was missing in a patient in Pakistan), the reported causes were septicemia with pneumonia (1), measles with seizures (1), pneumonia (1), seizures (1), and difficulty breathing (1). Three (50%) were inpatient, though one was discharged prior to death. The case fatality ratio was <0·1% in both countries.

**Fig. 3:**
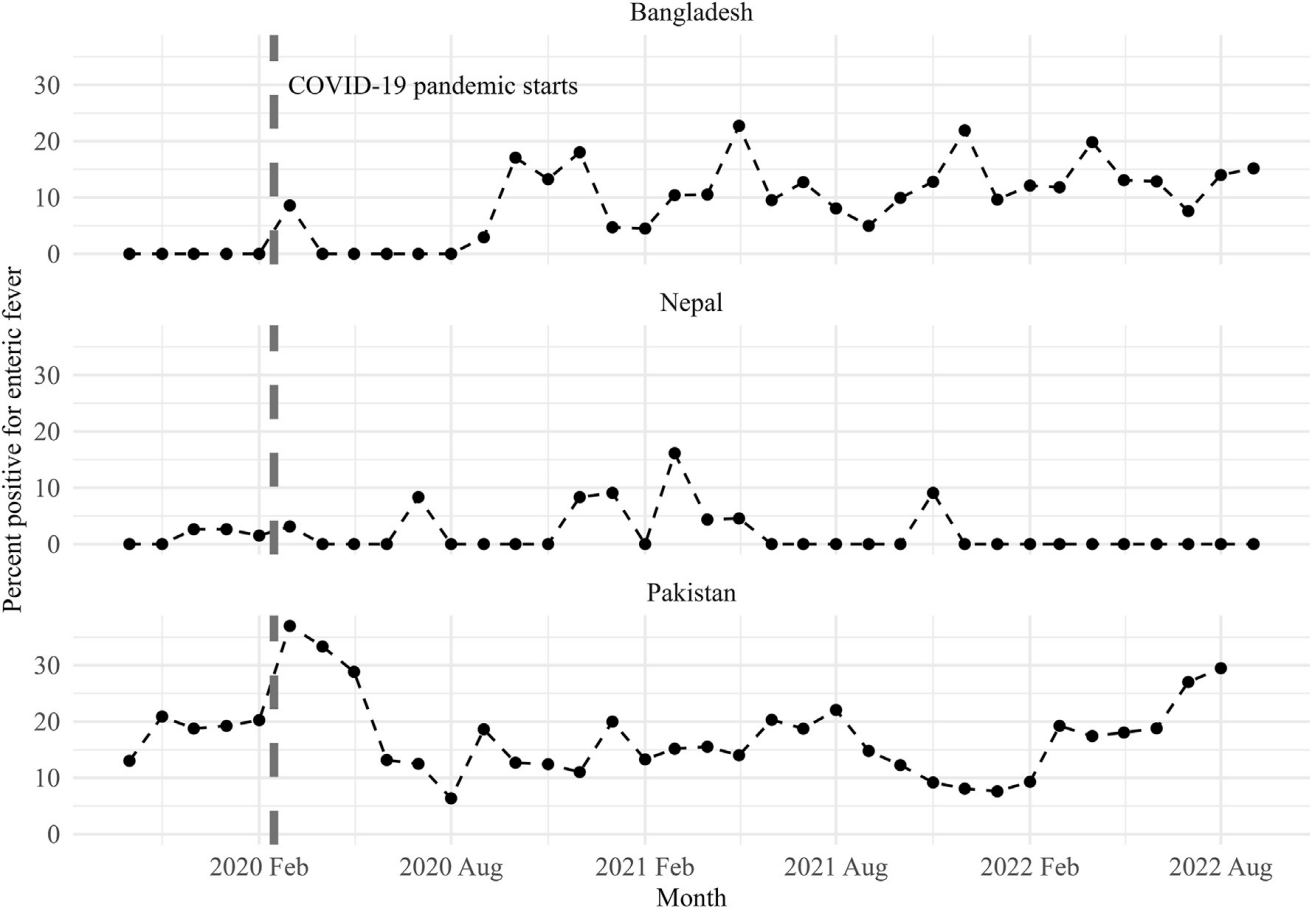
**Enteric fever positivity rate among participants enrolled prospectively**.

We measured trends in enteric fever positivity among prospectively enrolled participants. This calculation was limited to settings that collected data on participants prior to blood culture (Fig. 3). The average enteric fever positivity rate in Bangladesh was 8·6%, 1·9% in Nepal, and 17·4% in Pakistan. In Bangladesh, the positivity rate in late 2019 and the first half of 2020 were impacted by study procedures and COVID-19 changes. After processes stabilized, the positivity rate fluctuated. Pakistan had the highest positivity rate. However, towards the end of the study period, the positivity rate increased. In Nepal, positivity was largely stable at a very low level; most cases were enrolled retrospectively.

Most isolates (99·9% [8400/8410]) had antibiogram data available (Table 3). Resistance to antimicrobials varied across countries. Nearly all Typhi isolates in Bangladesh (98% [2183/2220]) and Pakistan (100% [5192/5205]) were resistant or non-susceptibility to a tested antimicrobial. In Nepal, 86% (89/104) were resistant or non-susceptible to at least one antimicrobial. Ampicillin resistance was not common in Bangladesh (20% [434/2220]), not found in Nepal, and very common in Pakistan (84% [4394/5200]). Azithromycin-resistant isolates were only detected in Bangladesh (2% [51/2220]). In Pakistan, resistance to third-generation cephalosporins was common; 76% of isolates were resistant to ceftriaxone (3921/5189) and cefixime (3929/5193). There were no isolates resistant to cephalosporins identified in Bangladesh or Nepal. In each country, the prevalence of *S.* Typhi resistance to two first-line antibiotics, chloramphenicol and cotrimoxazole, was similar across the two antibiotics (in Bangladesh: chloramphenicol 17% [376/2220], cotrimoxazole 17% [368/2200]; in Nepal, chloramphenicol 1% [1/103], cotrimoxazole 1% [1/103]; in Pakistan, chloramphenicol 82% [4232/5171], cotrimoxazole 81% [4227/5195]). A high prevalence of fluoroquinolone non-susceptibility was observed (Bangladesh, 98% [2160/2200]; Nepal, 86% [89/104]; Pakistan, 99% [5179/5195]) in the *S.* Typhi isolates. The prevalence of MDR typhoid was 16% (350/2200) in Bangladesh over the study period. There were no MDR isolates in Nepal. In Pakistan, 69% (3542/5158) of isolates were classified as XDR typhoid, and an additional 8·3% (431/5176) were classified as MDR, but not XDR, typhoid.

**Table 3:** Antibiotic resistance by country and pathogen.^a^

	Bangladesh		Nepal		Pakistan	
	n/N	%	n/N	%	n/N	%
***S.* Typhi**	**2220**		**104**		**5205**	
Any resistance or non-susceptibility	2183/2220	98%	89/104	86%	5192/5205	100%
Resistant to						
Ampicillin	434/2220	20%	0/94	0%	4394/5200	84%
Azithromycin	51/2220	2.3%	0/104	0%	0/4133	0%
Ceftriaxone	0/2200	0%	0/19	0%	3921/5189	76%
Cefixime	0/2200	0%	0/93	0%	3929/5193	76%
Chloramphenicol	376/2200	17%	1/103	1.0%	4232/5171	82%
Cotrimoxazole	368/2200	17%	1/103	1.0%	4227/5195	81%
Ciprofloxacin non-susceptibility	2160/2200	98%	89/104	86%	5179/5195	99%
Multidrug-resistant only^b^	350/2200	16%	0/103	0%	431/5176	8.3%
Extensively drug-resistant	0/2200	0%	0/104	0%	3542/5158	69%
***S.* Paratyphi A**	**382**		**16**		**473**	
Any resistance or non-susceptibility	382/382	100%	16/16	100%	468/473	99%
Resistant to						
Ampicillin	0/382	0%	0/13	0%	12/473	2.5%
Azithromycin	14/382	3.7%	0/16	0%	0/69	0%
Ceftriaxone	0/382	0%	0/1	0%	3/473	0.6%
Cefixime	0/382	0%	0/16	0%	3/473	0.6%
Chloramphenicol	0/382	0%	0/16	0%	0/469	0%
Cotrimoxazole	0/382	0%	0/16	0%	6/472	1.3%
Ciprofloxacin non-susceptibility	382/382	100%	16/16	100%	467/472	99%
Multidrug-resistant	0/382	0%	0/16	0%	0/473	0%

a Denominators vary based on number of specimens tested for each antibiotic.

b Excludes extensively drug-resistant specimens.

Resistance profiles of *S*. Paratyphi isolates differed from Typhi isolates. Nearly all were resistant or non-susceptible to at least one antibiotic in all countries (Bangladesh, 100% [382/382]; Nepal, 100% [16/16]; Pakistan, 99% [468/473]). Only isolates in Pakistan were resistant to ampicillin (2·5% [12/473]), cotrimoxazole (1·3% [6/472]), or any cephalosporin (0·6% [3/473]). Azithromycin resistance was observed in Bangladesh (3·7% [14/382]), but not in Nepal or Pakistan. No chloramphenicol-resistant paratyphoid isolates were detected in any of the sites. Almost every paratyphoid isolate was non-susceptible to fluoroquinolone in all three countries (Bangladesh, 100% [382/382]; Nepal, 100% [16/16]; Pakistan, 99% [467/472]). There were no MDR or XDR paratyphoid isolates.

## Discussion

We conducted prospective, healthcare facility-based enteric fever surveillance from 2019 to 2022 to describe the burden, illness severity, outcome, and AMR patterns in Dhaka, Bangladesh, Kathmandu Valley, Nepal, and Karachi, Pakistan. Our surveillance detected fewer cases of enteric fever during the first year of the pandemic than in prior years, though it is unclear how much of this change was due to less transmission of typhoid versus decreased health-seeking behavior. By summer 2022, case counts in all three locations were similar to pre-pandemic levels. In Pakistan, we did not see a marked reduction in cases after vaccine introduction. Additionally, transmission of multidrug-resistant and extensively–drug-resistant typhoid strains continued, but we did not observe widespread dissemination of pan-resistant strains.

The COVID-19 pandemic interfered significantly with study procedures. In Bangladesh, from March to September 2020, enrollment in the outpatient department and laboratory network sites was stopped. The team used remote or virtual options to enroll and follow-up a subset of cases during this period. The research activities in Nepal experienced multiple interruptions at both sites. Initially, from March–July 2020, study activity stopped at both active clinical surveillance sites in Nepal following a nationwide lockdown. Study activities resumed and were again halted several times. In Pakistan, all hospital recruitment stopped in March 2020. Activities resumed in both main hospitals in May 2020, and for one outpatient department, in August 2020. These disruptions impacted ongoing prospective recruitment for the study.

The lower frequency of typhoid and paratyphoid we observed during the COVID-19 pandemic may have several causes. First, lockdowns prevented or restricted study screening and enrollment. Many patients avoided healthcare settings, both due to lockdown as well as fear of SARS-CoV-2 transmission in healthcare settings.^18^ Second, study sites changed their blood culture practices and patient pathways; patients with fever who would be otherwise eligible for this study may have been routed for SARS-CoV-2 testing, with other testing reduced to minimize exposure to potential COVID-19. Third, overall infection transmission and disease from enteric fever may have truly decreased as a result of travel restrictions; closures of restaurants, bars, and street food vendors; and improvement in hygiene practices.

Trends during the pandemic did differ by country. In 2020 and 2021, Bangladesh experienced a significant decline in confirmed enteric fever cases, followed by an upward trend from 2022. Nepal witnessed a similar decline during the early COVID-19 period and the case count and positivity rate did not rebound during the study period. Changes in eating outside the house and hygiene practices (in response to both the pandemic and to a local cholera outbreak) likely contributed to the lower burden observed.^21^ Another study also reported a decline in *Salmonella* and other enteric infections when comparing the periods before and after the COVID-19 pandemic.^22^

We observed the highest number of cases and enteric fever positivity in Pakistan during the study period. Case counts exceeded pre-pandemic case counts throughout 2022. These later months of high transmission reflect a period of intensified population movements following flooding in Sindh province, where Karachi is located.^23^ This high transmission occurred despite the introduction of TCV in Karachi, Pakistan (where this study took place), in November 2019, at the beginning of the study period. Despite this campaign, we observed a large burden of typhoid in vaccine-eligible children, few of whom reported receipt of TCV. Administrative coverage levels show more than 95% of eligible children were vaccinated.^24^ Our findings suggest either the vaccine is less effective than demonstrated in multiple trials or, more likely, that administrative coverage data does not fully reflect population coverage. A better understanding of overall vaccine coverage would help officials identify and intervene in areas with insufficient TCV coverage. Additionally, strengthening routine immunization coverage, targeted catch-up campaigns in areas not covered in the initial campaign, and addressing vaccine hesitancy could help Pakistan achieve the full benefits of TCV introduction.

Nepal also introduced TCV, though at the end of the study period; we did not have enough data after introduction to assess potential changes due to vaccination. Additionally, the vaccine was introduced during a period of low typhoid transmission overall. Long-term surveillance and other research could help determine the impact of vaccination in the country, though are unlikely to fully distinguish if this lowered case burden resulted from vaccination or changes in transmission pathways.

This study also reported antimicrobial resistance for typhoid and paratyphoid. Resistance patterns in this period were similar to those observed in the same locations between 2016 and 2019.^25^ In Bangladesh, we found the most azithromycin-resistant isolates, which has been described elsewhere.^26^ In Pakistan, MDR and XDR typhoid remain alarmingly common and increase the risk of treatment failure.^27^ Recently, a patient with carbapenem-resistant typhoid was reported in Pakistan.^27^ Carbapenem resistance in typhoid would significantly limit treatment options. Additionally, fluoroquinolone non-susceptibility is widespread among typhoid isolates globally, with high levels of resistance observed across Bangladesh, Nepal and Pakistan. This situation is compounded by the potential emergence of extensively-drug resistant typhoid strains with azithromycin resistance, which pose a worrying threat to public health.^28^^,^^29^

Our study is subject to several limitations. We describe the number of patients with enteric fever who presented to care at our sentinel sites, which represents only a small portion of the overall burden of enteric fever in each location. Case count varies both by burden of enteric fever and likelihood of seeking care, and it is difficult to distinguish which factor drives the changes in case count. This limitation was compounded due to the COVID-19 pandemic, particularly early on; care-seeking behavior changed as individual perception of risk and necessary care changed. Additionally, hospital closures and consequent changes in study implementation modified our measurement procedures. Changes in hospital procedure for febrile patients may have routed potential participants away from prospective recruitment in the study, especially early in the pandemic. During early lockdowns or periods of intensified transmission, febrile patients may have received different blood culture recommendations compared to participants outside these time periods. However, all participants with blood cultures positive for enteric fever at study facilities would be eligible for study enrollment. Additionally, we may have missed cases of enteric fever due to blood culture sensitivity, particularly among patients who previously used antibiotics, which varies between countries, age groups, and over time within countries. Finally, patient medical history and follow-up data, including typhoid vaccination status, are self-reported; these data may reflect recall or social desirability biases. In Bangladesh, only a randomly selected 25% of typhoid patients (and all paratyphoid patients) were called for follow-up interviews six weeks after their illness. While follow-up data is only available for this subset, we expect any differences between the observed and unobserved to be at random.

In conclusion, even during the COVID-19 pandemic, we observed a persistently high burden of typhoid within the SEAP study sites in Pakistan, Nepal, and Bangladesh. Ongoing surveillance provides insights into the burden of disease in three countries at different TCV introduction stages. Continued data collection before, during, and after TCV introduction holds promise to inform further next steps, such as the need for broader access to vaccination, the impact of vaccination on antimicrobial resistance, and the duration of vaccine protection.

## Contributors

Conceptualization and funding acquisition: MTY, JRA, SPL, DOG, FNQ, SKS, SS; Data curation, visualization, and analysis: SRN, IFD, KD, NK, APA, KI, SI, MSI, MAM, MTY, JCS; Investigation, supervision, and project administration: SJM, SRN, IFD, KD, MTY, DT, APA, ASC, SD, KI, SI, MSI, TK, MAM, MHR, NSS, JCS, RS, JRA, SPL, DOG, FNQ, SKS, SS, HS, ABJ, SN; Resources: DCD; Validation: SJM, SRN, KD, NK, DT, ASC, MSI, JCS, RS, JRA, SPL, DOG, SS; Writing original draft: SJM, SRN, IFD, KD, NK, MTY, DT.

Data was accessed and verified by SJM, SRN, IFD, KD, NK, MTY, and DT. All authors reviewed the manuscript, provided critical inputs and approved the final manuscript. The decision to submit this manuscript was made by SJM, SRN, IFD, KD, JRA, SPL, DOG, FNQ, SKS, and SS.

## Data sharing statement

De-identified study data can be shared upon request by other investigators and review. Data requests can be sent to the corresponding author.

## Declaration of interests

The authors declare no competing interests.
