# Supplementary material for: Changes in enteric fever trends during the COVID-19 pandemic from the Surveillance for Enteric Fever in Asia Project: a cross-sectional study
Source: Lancet Reg Health Southeast Asia. 2025 Mar 29;35:100562. doi: 10.1016/j.lansea.2025.100562 (PMC11995784; doi:10.1016/j.lansea.2025.100562)
Supplement: Appendix [file mmc1.docx]

**Appendix**

1. List of facilities participating in SEAP surveillance, October 2019–September 2022.

| **Country** | **Facility** | **Enrollment type** | **Start** | **End** |
| --- | --- | --- | --- | --- |
| Bangladesh | Dhaka Shishu Hospital | Hospital-based | 10/1/2019 | 9/30/2022 |
| Bangladesh | Shishu Sasthya Foundation Hospital | Hospital-based | 10/1/2019 | 9/30/2022 |
| Bangladesh | Popular Diagnostic Center - Dhanmondi | Laboratory network | 10/1/2019 | 9/30/2022 |
| Bangladesh | Popular Diagnostic Center - Mirpur | Laboratory network | 10/1/2019 | 9/30/2022 |
| Bangladesh | Popular Diagnostic Center - Shamoly | Laboratory network | 10/1/2019 | 9/30/2022 |
| Nepal | Dhulikhel Hospital | Hospital-based | 10/1/2019 | 9/30/2022 |
| Nepal | Kathmandu Model Hospital | Hospital-based | 10/1/2019 | 9/30/2022 |
| Nepal | Alka Hospital | Laboratory network | 10/1/2019 | 6/30/2020 |
| Nepal | Bir Hospital | Laboratory network | 10/1/2019 | 9/30/2022 |
| Nepal | Helping Hands Clinic | Hospital-based | 10/1/2019 | 9/30/2022 |
| Nepal | Kathmandu Medical College and Teaching Hospital | Laboratory network | 10/1/2019 | 12/2/2020 |
| Nepal | Nepal Medical College | Laboratory network | 10/1/2019 | 9/30/2022 |
| Pakistan | Aga Khan University Hospital | Hospital-based | 10/1/2019 | 8/15/2022 |
| Pakistan | Kharadar General Hospital | Hospital-based | 10/1/2019 | 8/15/2022 |
| Pakistan | Kharadar General Hospital Laboratory and Laboratory Network | Laboratory network | 10/1/2019 | 8/15/2022 |
